# Dyskinesia and brain-derived neurotrophic factor levels after long-term levodopa and nicotinic receptor agonist treatments in female mice with near-total unilateral dopaminergic denervation

**DOI:** 10.1186/s12868-018-0478-0

**Published:** 2018-11-29

**Authors:** Sakari Leino, Samuel Kohtala, Tomi Rantamäki, Sini K. Koski, Saara Rannanpää, Outi Salminen

**Affiliations:** 1 Division of Pharmacology and Pharmacotherapy, Faculty of Pharmacy, University of Helsinki, P.O. Box 56, 00014 Helsinki, Finland; 2 Laboratory of Neurotherapeutics, Division of Physiology and Neuroscience, Department of Biosciences, University of Helsinki, 00014 Helsinki, Finland

**Keywords:** Parkinson’s disease, Levodopa-induced dyskinesia, BDNF, Nicotine, Alpha7 nicotinic receptors

## Abstract

**Background:**

The treatment of Parkinson’s disease is often complicated by levodopa-induced dyskinesia (LID). Nicotinic acetylcholine receptor agonists can alleviate LID in animal models but may be less effective in conditions of severe dopaminergic denervation. While the mechanisms of LID remain incompletely understood, elevated corticostriatal levels of the brain-derived neurotrophic factor (BDNF) have been suggested to play a role. Here, female mice with near-total unilateral 6-hydroxydopamine-induced nigrostriatal lesions were chronically treated with levodopa, and the effects of the α7 nicotinic receptor partial agonist AZD0328 and nicotine on LID were assessed. At the end of the experiment, BDNF protein levels in the prefrontal cortex and striatum were measured.

**Results:**

Five-day treatments with three escalating doses of AZD0328 and a 10-week treatment with nicotine failed to alleviate LID. BDNF levels in the lesioned striatum correlated positively with LID severity, but no evidence was found for a levodopa-induced elevation of corticostriatal BDNF in the lesioned hemisphere. The nicotine treatment decreased BDNF levels in the prefrontal cortex but had no effect on striatal BDNF.

**Conclusions:**

The findings suggest that treatment of LID with nicotinic agonists may lose its effectiveness as the disease progresses, represent further evidence for a role for BDNF in LID, and expand previous knowledge on the effects of long-term nicotine treatment on BDNF.

**Electronic supplementary material:**

The online version of this article (10.1186/s12868-018-0478-0) contains supplementary material, which is available to authorized users.

## Background

The primary cause of the motor symptoms of Parkinson’s disease is a lack of dopamine in the striatum resulting from a loss of dopaminergic neurons in the substantia nigra pars compacta (SNC) [1]. Replenishment of the lost dopamine with the precursor molecule levodopa (l-3,4-dihydroxyphenylalanine) usually provides symptomatic relief [1]. However, complications, including abnormal involuntary movements termed levodopa-induced dyskinesia (LID), often appear as the treatment is continued [2].

While current treatment options against LID remain suboptimal, including only the multi-target drug amantadine and invasive deep-brain stimulation, preclinical studies have revealed many potential treatment targets [2]. These include neuronal nicotinic acetylcholine receptors (nAChRs), widely expressed ion channel receptors that modulate most neurotransmission in the brain, including nigrostriatal dopaminergic neurotransmission [3, 4]. Extensive preclinical as well as clinical studies have described beneficial effects by nAChR ligands on cognition and mood, and preclinical studies have also extensively investigated nAChR-mediated neuroprotective effects in the context of nigrostriatal denervation [5]. Furthermore, chronic treatment with both selective and non-selective nAChR agonists has been shown to alleviate LID in multiple animal models [5]. While the specific mechanisms of these antidyskinetic effects are not completely understood, they are hypothesized to be mediated by receptor desensitization [5]. In some studies the most effective alleviation of LID has been observed in conditions of only partial dopaminergic denervation [6–8], suggesting that the degree of remaining nigrostriatal innervation may be an important determinant of treatment effectiveness.

The pathophysiology of LID has also been the focus of many studies and involves numerous mechanisms, brain areas and neurotransmitters [2]. Aberrant synaptic plasticity has been suggested to underlie LID, and rodent studies have demonstrated that LID is associated with a loss of bidirectional synaptic plasticity in striatal output pathways [9–12]. One important regulator of synaptic plasticity is the brain-derived neurotrophic factor (BDNF) [13, 14], raising the hypothesis that changes in BDNF levels or function would be associated with LID. Indeed, polymorphisms in the *Bdnf* gene have been linked to increased risk of LID in Parkinson’s disease patients [15, 16], and striatal overexpression of BDNF increases susceptibility to LID in hemiparkinsonian rodents [17]. Furthermore, increases in cortical and striatal *Bdnf* mRNA were observed in hemiparkinsonian rodents after short-term levodopa administration and, in the case of the frontal cortex and the striatum, were found to be especially prominent in the lesioned brain hemisphere [18, 19]. Thus, elevated corticostriatal BDNF was suggested to account for LID [18].

In this study, we utilized mice with near-total unilateral 6-hydroxydopamine (6-OHDA) lesions of the dopaminergic nigrostriatal pathway, made possible by intensive postoperative care allowing the survival of even the most severely lesioned animals, to study whether long-term treatment with nAChR agonists can alleviate LID in conditions of severe unilateral dopaminergic denervation. The specific nAChR agonists used were the selective α7 receptor agonist AZD0328 [20] and the prototypical non-selective agonist nicotine. As LID can be associated with changes in BDNF, we also investigated the effects of the long-term levodopa and nicotine treatments on the BDNF protein in the striatum and prefrontal cortex (PFC). A frontal cortical area was chosen as denervation-specific changes in *Bdnf* mRNA were previously observed in the frontal but not the motor-premotor cortices [18, 19].

## Results

**Fig. 1:**
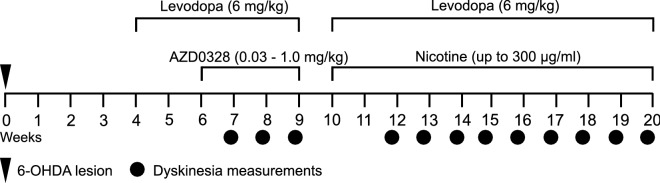
Timeline of the experiments. Mice were unilaterally lesioned with 6-hydroxydopamine (6-OHDA) injections into the medial forebrain bundle. Levodopa (6 mg/kg) together with benserazide (1.5 mg/kg) was administered s.c. daily (Mon–Fri). The α7 nicotinic receptor partial agonist AZD0328 was administered s.c. at three escalating doses for 1 week (Mon–Fri) per dose. Nicotine was administered continuously in saccharin-sweetened drinking water, with the concentration gradually increased to 300 µg/ml over 2 weeks. Severity of levodopa-induced dyskinesia was assessed from videos recorded weekly at the indicated time points

**Fig. 2:**
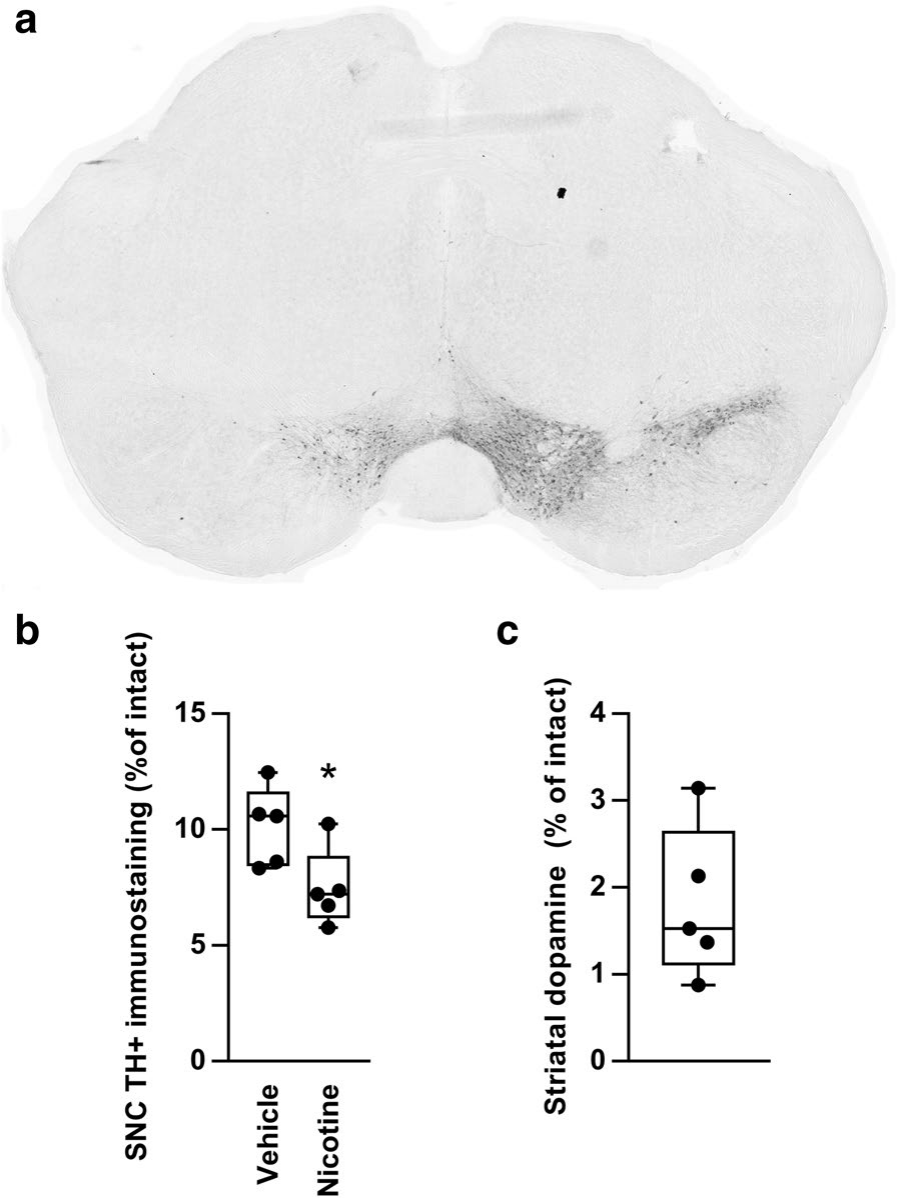
Characterization of the near-total unilateral nigrostriatal lesion model. **a** Representative section, immunostained for tyrosine hydroxylase, showing the ventral midbrain dopaminergic areas, including the substantia nigra pars compacta (SNC), of a study animal lesioned with a 6-hydroxydopamine injection into the medial forebrain bundle. **b** Tyrosine hydroxylase (TH)-positive immunostaining remaining in the SNC of the study animals at the end of the experiments (*n* = 10). Less immunostaining remained in animals treated chronically with nicotine. **c** Dopamine remaining in the dorsal striatum of a separate set of animals, lesioned with identical 6-hydroxydopamine injections, as measured with HPLC (*n *=5). *Difference to vehicle, *P *< 0.05, *t* test

See Fig. 1 for the study timeline and Fig. 2 for data characterizing the nigrostriatal lesion model. The study mice, unilaterally lesioned with 6-OHDA injections into the medial forebrain bundle (MFB), had suffered a severe loss of tyrosine hydroxylase (TH)-positive immunostaining in the lesioned SNC, with 8.8 ± 0.7% of immunostaining remaining at the end of the experiments (*n* = 10). More immunostaining remained in vehicle-treated than in nicotine-treated animals (t(8) = 2.51, *P *= 0.037). Striatal dopaminergic denervation could not be assessed in the study animals as striatal samples were used for the BDNF assay, but in a separate experiment female C57BL/6J mice lesioned with identical 6-OHDA injections had suffered a near-total loss of dopamine in the lesioned dorsal striatum, as measured by high-performance liquid chromatography (HPLC), with only 1.8 ± 0.4% of dopamine remaining (*n *= 5).

**Fig. 3:**
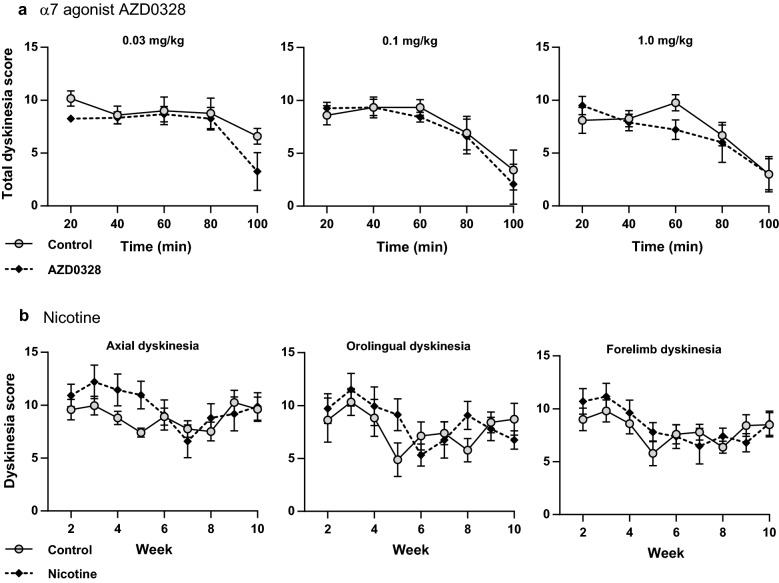
No effect on levodopa-induced dyskinesia by chronic treatment with nicotinic receptor agonists. Mice were lesioned unilaterally with 6-OHDA, and dyskinesia was pre-induced by 2 weeks of levodopa treatment (6 mg/kg s.c.). **a** The α7 receptor partial agonist AZD0328 was administered s.c. 30 min before levodopa for 1 week per dose (5 days per week), and dyskinesia severity was assessed from video recordings captured on the fifth day. No statistically significant differences in total dyskinesia severity between treatment groups were found. **b** After a 1-week washout, chronic levodopa treatment (6 mg/kg) was continued and nicotine treatment in drinking water (up to 300 µg/ml) was initiated. Dyskinesia severity was assessed from weekly video recordings. Nicotine treatment did not reduce but transiently exacerbated dyskinesia severity, with a statistically significant effect on axial dyskinesia (two-way repeated measures ANOVA, treatment × time, *P *< 0.05). Values represent the mean ± SEM of 6 mice

Dyskinesia was induced in lesioned animals with daily (5 days per week) levodopa (6 mg/kg) and benserazide (1.5 mg/kg) s.c. injections. Treatment with escalating doses of the α7 partial agonist AZD0328 for 5 days (Mon–Fri) per dose failed to reduce total dyskinesia (Fig. 3a). When LID subtypes were analyzed separately (data in Additional file 1), only one statistically significant result was observed, where a reduction in axial dyskinesia was observed with the lowest dose of 0.03 mg/kg at the last measuring time point (100 min) (*n* = 6 per group; treatment × time interaction, F(4,40) = 3.37, *P* = 0.018).

During treatment with nicotine in drinking water, the estimated average daily intake of nicotine at the highest concentration was 35.3 ± 9.6 mg/kg, nearly identical to our previous study where decreased LID was observed in partially lesioned nicotine-treated mice [21]. In the present study, however, the nicotine treatment failed to reduce LID in mice with near-total unilateral lesions (Fig. 3b). On the contrary, the nicotine treatment appeared to transiently exacerbate dyskinesia during the first weeks of the treatment, with a statistically significant effect on axial dyskinesia (*n* = 6 per group; treatment × time interactions: axial dyskinesia, F(8,80) = 3.04, *P* = 0.005; forelimb dyskinesia, F(8,80) = 1.93, *P* = 0.067; orolingual dyskinesia, F(2.7,27.2) = 2.09, *P *= 0.13). No multiple comparisons tests reached statistical significance.

**Fig. 4:**
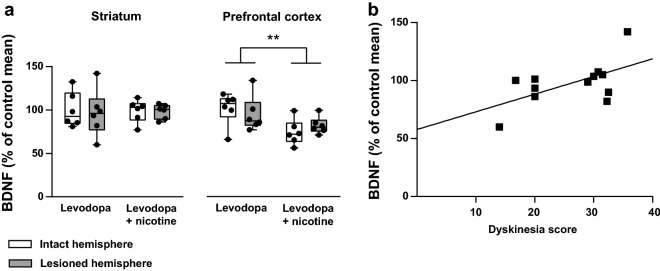
Brain-derived neurotrophic factor (BDNF) protein levels in chronically drug-treated lesioned mice. **a** No interhemispheric differences in BDNF protein levels of the striatum and the prefrontal cortex were found after 15 weeks of levodopa treatment. After 10 weeks of chronic nicotine treatment BDNF levels were reduced in the prefrontal cortex of nicotine-treated animals, with no effect on striatal BDNF. Plots show median, quartiles, range, and distribution (*n* = 6 per group). **Main effect of treatment *P* < 0.01, two-way ANOVA. **b** BDNF levels in the lesioned striatum were positively correlated with dyskinesia severity (*r* = 0.582, *P* < 0.05, *n* = 12)

At the end of the experiments with nAChR agonists, BDNF protein levels in the striatum and PFC were measured (Fig. 4). No difference in BDNF levels between the lesioned and intact hemispheres was observed in either brain area. Nicotine treatment had no effect on striatal BDNF. BDNF levels in the PFC, however, were decreased in nicotine-treated animals (*n* = 6 per group; main effect of treatment: F(1,20) = 9.06, *P *= 0.007). For analysis of correlation between striatal BDNF and dyskinesia severity, control and nicotine groups were combined as neither dyskinesia severity at the end of the experiment nor striatal BDNF levels differed between groups. BDNF levels in the lesioned striatum had a positive linear correlation with total dyskinesia scores at the time of the last dyskinesia measurement (*n* = 12, *r* = 0.582, *P *= 0.047). No other statistically significant correlations between dyskinesia scores and BDNF levels were found (data in Additional file 1).

## Discussion

Nicotinic acetylcholine receptor agonists can alleviate LID in animal models of Parkinson’s disease, as shown by Quik and colleagues [5] as well as by us [21]. However, the extent of dopaminergic denervation may be important in determining treatment effectiveness. Indeed, in the present study, mice with near-total unilateral nigrostriatal lesions were treated with the α7 nAChR agonist AZD0328 and nicotine without any reduction in LID. The finding is partially in line with previous rodent and monkey studies, where the most severely lesioned animals were typically found to be less responsive or even completely unresponsive to antidyskinetic nicotine treatment [6–8, 22]. The present finding suggests that a partially intact nigrostriatal pathway may be required or at least highly conducive for the treatment of LID with nicotine, and that the treatment may thus lose its effectiveness as the disease progresses.

Nigrostriatal dopaminergic neurons express a large variety of both somatodendritic and presynaptic nAChRs that modulate striatal dopaminergic neurotransmission [3]. Given the critical importance of striatal dopamine for LID [2], these nAChRs stand out as obvious candidates for mediators of the antidyskinetic effects by nAChR agonists. Indeed, previous studies have suggested an association between antidyskinetic effects and a dampening of nAChR-mediated striatal dopamine release [21, 23]. As these nAChRs are mostly lost in a severe nigrostriatal lesion, a requirement of partially intact nigrostriatal innervation for robust effects by nicotine would not be surprising.

In our previous study, we demonstrated that chronic nicotine treatment in drinking water had an antidyskinetic effect in mice with partial unilateral nigrostriatal lesions [21]. The previous study utilized the intrastriatal 6-OHDA lesion model, characterized by less severe and topographically more restricted dopaminergic denervation and by LID that is less severe and requires much higher levodopa dosages to develop [21, 24]. In our two studies, nicotine was administered using the same concentration and a similar treatment length, and the average daily intake of nicotine was practically identical. However, the two studies differed in the timing of the initiation of drug treatments: in the previous study, the nicotine and levodopa treatments were initiated simultaneously in naïve animals, while in the present study pre-established LID was treated with nicotine.

Previous mouse studies have consistently reported antidyskinetic effects by nicotine also on pre-established LID [7, 8, 25]. However, dyskinesia induced by the somewhat high levodopa dose used in the present study (6 mg/kg) was previously studied only in experiments using nicotine pretreatment [7]. As dopaminergic denervation is the main contributor to sensitization to levodopa and LID [2], in the present study the combination of near-total unilateral dopaminergic denervation and a high levodopa dose may have led to deeply ingrained LID resistant to attenuation by later nicotine treatment. Simultaneously, the potential antidyskinetic effects of the treatment may have been blunted by the practically total unilateral loss of nAChRs expressed on nigrostriatal neurons. This situation could be comparable to starting nicotine treatment in advanced PD associated with a high levodopa dose regime and existing severe LID. Thus, it may turn out to be necessary to begin potential clinical antidyskinetic treatments with nAChR agonists as early as possible.

Another notable difference is that desipramine pretreatment was used to inhibit noradrenergic neurodegeneration in the previous study but not in the present study. In rats lesioned with intra-MFB 6-OHDA injections, desipramine pretreatment has resulted in both decreased and increased LID [26, 27]. Thus, it is unclear what role the desipramine pretreatment may have had in the expression of LID in our studies. However, as none of the previous rodent studies on antidyskinetic effects of nAChR agonists used desipramine [6–8, 25, 28], it is unlikely that the lack of desipramine pretreatment would explain the lack of efficacy of nicotine in the present study.

It is noteworthy that a previous mouse study, utilizing very similar methods of lesioning, LID induction, and chronic nicotine treatment, did observe somewhat inhibited LID even when dopamine transporter levels of the lesioned striatum were determined to be less than 1% of intact levels [7]. While the reason for this discrepancy is unclear, it suggests that the sparing of nigrostriatal nAChRs may indeed be conducive but not strictly necessary for antidyskinetic effects. Note also that the nicotine treatment did have a statistically significant (although not alleviating) effect on axial LID also in the present study. Thus, also other nAChR populations than those expressed on dopaminergic neurons are likely to contribute to the modulation of LID by nAChR ligands. The identity of these receptor populations has not so far been directly investigated, but they could be potential drug targets in the treatment of LID associated with advanced PD.

To our knowledge, the antidyskinetic potential of α7 nAChR agonism has not been previously studied in rodents, but monkey studies have reported alleviation of LID by various α7 agonists [29–31]. Although AZD0328 was here administered for only 3 weeks, monkey studies have shown significant effects already within 1–2 weeks [29–31]. Furthermore, comparable AZD0328 doses as used here were previously shown in mice to result in various behavioral effects, changes in dopamine release, and (at the highest dose) maximal brain α7 receptor occupancy [20, 32]. These previous findings suggest that a too short treatment time or too low dosages are unlikely to explain the present lack of efficacy. Thus, also the lack of an antidyskinetic effect by AZD0328 may be explained by the near-total unilateral lesion. Note that while α7 agonism was reported in monkeys to reduce also LID associated with relatively severe nigrostriatal damage, the loss of striatal innervation was not total [30]. Alternatively, α7 agonism may not be antidyskinetic in mice in the first place. Note that dyskinetic α7-knockout mice respond to nicotine treatment similarly to wild-type mice [8], suggesting that in mice α7 agonism does not have a major role in nicotine’s antidyskinetic effects. It should also be noted that a recent phase 2 clinical study observed no significant reduction in LID or parkinsonism after 4 weeks of treatment with an α7 receptor partial agonist [33].

Based on findings of increased *Bdnf* mRNA in the lesioned brain hemisphere after levodopa administration in hemiparkinsonian rats, elevated BDNF in corticostriatal neurons has been suggested to underlie levodopa-induced abnormal movements [18]. In the present study, BDNF protein levels in the lesioned striatum and LID severity were positively correlated, similar to a previously reported correlation between striatal *Bdnf* mRNA and LID severity in rats [19]. However, no differences in BDNF protein levels between the intact and lesioned hemispheres were observed, in contrast to earlier findings on cortical and striatal *Bdnf* mRNA [18, 19]. The contrasting results may be due to the different targets of measurement (protein vs. mRNA), or be explained by the considerably longer duration of levodopa treatment (months vs. days/weeks).

The observed correlation between BDNF and LID represents further evidence for a role for BDNF in LID pathophysiology, demonstrating that the previously reported mRNA level correlation is also present when measuring the protein, a relationship which is not always predictable in the case of BDNF [34]. However, the present findings do not directly support the hypothesis that LID is caused by a levodopa-induced elevation of corticostriatal BDNF that is further enhanced in conditions of dopaminergic denervation [18]. It does remain possible that such an elevation, observable in a unilateral animal model as an interhemispheric difference, would reflect the initial sensitization to levodopa and thus disappear after the early phase of the treatment. Note also that while striatal BDNF protein is mainly derived from corticostriatal glutamatergic afferents, nigrostriatal dopamine terminals do represent a minor source of striatal BDNF [35]. Unlike in the present study, a near-total unilateral depletion of striatal dopamine in 6-OHDA-lesioned rats was associated with a modest (14%) decrease in striatal BDNF protein levels [35]. While the reason for this discrepancy is unclear, opposite changes in striatal BDNF by the nigrostriatal lesion and the levodopa treatment may in principle have occurred.

The nicotine treatment had no effect on striatal BDNF levels, in line with a previous study on long-term nicotine administration via drinking water [36]. Other studies have, however, reported both increases and decreases in striatal BDNF after chronic nicotine administration via various routes [37–39]. In the present study, nicotine treatment via drinking water significantly decreased BDNF levels in the prefrontal cortex, similarly as in a previous study on rat frontal cortex utilizing repeated subcutaneous nicotine injections [40]. While the previous study hypothesized that the nicotine-induced decrease in cortical BDNF was related to a withdrawal stress response, the present study included no withdrawal period, making this explanation unlikely. Note also that in another rat study a nicotine-induced increase in cortical BDNF was reported [41]. All in all, the effects of chronic nicotine treatment on BDNF appear highly variable and possibly dependent on issues such as the route of administration.

Finally, although nicotine has neuroprotective effects [5], in the present study the nicotine-treated animals in fact had less TH-positive immunostaining remaining in the SNC than the control animals. The observation of no neurorestoration is in line with a previous study showing that nicotine does not have neurorestorative effects in rats or monkeys when administered after lesioning [42]. The reason for less TH remaining in the nicotine-treated animals is unclear. However, note that as the animals were randomly re-divided to nicotine and vehicle groups after AZD0328 treatment and a washout, the previous α7 agonist treatment could in principle confound these results.

## Conclusions

We show here in hemiparkinsonian mice that levodopa-induced dyskinesia associated with near-total unilateral nigrostriatal dopaminergic denervation can be unresponsive to treatment with nAChR agonists. The results suggest that a partially intact nigrostriatal pathway is highly conducive for nAChR-mediated antidyskinetic drug effects and that treatment of LID with nAChR agonists may lose its effectiveness as the disease progresses. These findings may be important to account for when drafting potential future strategies for the treatment of late-stage Parkinson’s disease with nAChR ligands. In addition, we show that BDNF protein levels in the lesioned striatum are correlated with LID severity, representing further evidence for a role for BDNF in LID. However, no evidence was found for a levodopa-induced elevation in corticostriatal BDNF levels of the lesioned hemisphere, suggesting that such elevations observed previously could reflect initial sensitization. The present findings also expand previous knowledge on the effects of long-term nicotine treatment on BDNF. These findings could help steer the planning of future studies investigating the aberrant plasticity that is thought to underlie LID.

## Methods

### Animals

Adult female C57BL/6J mice (Envigo Netherlands), aged 28 weeks and weighing 23–31 g at the beginning of the study, were group-housed in a temperature- and humidity-controlled environment under a 12 h light/dark cycle. Aged and thus relatively weighty mice were used to lessen the impact of postoperative weight loss. Only female mice were used as male mice are prone to suffer penile prolapse complications after a severe 6-OHDA lesion [43]. All experiments were conducted following local and EU laws and regulations and authorized by the national Animal Experiment Board of Finland.

### Drugs

Benserazide hydrochloride, 6-hydroxydopamine hydrochloride, levodopa methyl ester hydrochloride and (–)-nicotine were purchased from Sigma-Aldrich (St. Louis, MO, USA). AZD0328 ((2´R)-spiro-[1-azabicyclo[2.2.2]octane-3,2´(3´H)-furo[2,3-b]pyridine] D-tartrate) was provided free of charge by AstraZeneca (Cambridge, MA, USA). Solid drugs were dissolved in saline and administered at a volume of 10 ml/kg. Solutions of lidocaine (Orion Pharma, Finland), buprenorphine (Temgesic, RB Pharmaceuticals, UK), and carprofen (Rimadyl Vet, Pfizer Animal Health, Finland) were diluted in saline. The doses of drugs refer to their free bases.

### Unilateral 6-OHDA lesion and postoperative care

Stereotactic surgeries were conducted under isoflurane anesthesia (4% induction, 2% maintenance) following the description of Thiele et al. [43]. In brief, 6-OHDA (3 µg in 0.2 µl of 0.02% ascorbate-saline) was injected into the right MFB at the following coordinates relative to the bregma or the dural surface: A/P − 1.2 mm; L/M − 1.1 mm; D/V − 5.0 mm. Lidocaine, buprenorphine (0.1 mg/kg, s.c. once before surgery), and carprofen (5 mg/kg, s.c. once after surgery) were used for pain relief. Intensive postoperative care included warm saline injections to prevent dehydration, alleviating hypothermia with heating pads, food pellets softened by soaking in water, high-energy palatable diet (Bacon Softies, Bio-Serv, Flemington, NJ, USA; Nutriplus gel, Virbac, Carros, France), and feeding by hand. One animal out of 15 was euthanized due to a poor postoperative condition (postoperative mortality 7%).

### Drug treatments

The timeline of the experiments is given in Fig. 1. Levodopa (6 mg/kg) and benserazide (1.5 mg/kg) were administered daily (Mon–Fri) as a single s.c. injection. A relatively high but not excessive levodopa dose was chosen on the basis of literature [24, 43, 44] and our previous experience to ensure robust development of all types of dyskinetic behaviors in the majority of animals. Two mice, however, did not develop LID and were removed from the study before the nAChR agonist treatments. After 2 weeks of levodopa administration, mice were randomly assigned to two treatment groups (*n* = 6). While continuing the levodopa treatment, one group was also treated with the α7 partial agonist AZD0328 [32] at escalating doses of 0.03 mg/kg, 0.1 mg/kg, and 1.0 mg/kg, for 1 week per dose (5 days per week, Mon–Fri), administered s.c. 30 min prior to levodopa. The control group received saline injections. Dyskinesia severity was assessed weekly on the fifth day of administration.

After a 1-week washout (effects of AZD0328 on behavior and receptor expression disappear by 72 h [32]), mice were randomly re-divided into two treatment groups (*n* = 6). Levodopa treatment was then resumed, and nicotine treatment in saccharin-sweetened drinking water (up to 300 µg/ml) was initiated as described previously [7]. The concentration of 300 µg/ml was chosen as it is the concentration used in previous studies on LID in 6-OHDA-lesioned mice [7, 8, 21] and as higher concentrations do not result in higher nicotine intake due to reduced fluid intake [45]. Previous studies in our lab have shown that a similar method of administration results in mean brain concentrations of nicotine ranging from 243 to 329 ng/g, sufficient to induce nAChR desensitization [46]. Treatment with levodopa and nicotine was continued for 10 weeks in total with weekly assessments of dyskinesia severity, after which the animals were killed and the brains collected for biochemical analyses.

### Assessment of dyskinesia severity

Following the levodopa injection, the behavior of the mouse was video-recorded every 20 min for 60 s over a 100 min period in a transparent cylinder flanked by two vertical mirrors. Dyskinesia severity was assessed from the recordings by a person blinded to the treatment condition using previously described scoring criteria [21]. Briefly, dyskinetic behaviors were divided into three subtypes (axial, orolingual, and forelimb) and a score ranging from 0 to 4 was assigned for each subtype based on dyskinesia frequency and amplitude.

### BDNF protein analysis

BDNF protein levels in the striatum and the PFC were determined 1 week after the last dyskinesia measurement. Levodopa and nicotine treatments were continued until the mice were killed by cervical dislocation 4 h after the last levodopa administration and the brain dissected on ice. Samples were stored at -80 °C until use. Extraction of proteins was done essentially as previously described [47]. A commercial ELISA kit (BDNF Emax ImmunoAssay; Promega, Madison, WI, USA) was used for the BDNF analyses. The manufacturer’s instructions were followed except for two modifications. First, Optacoat (ALerCHEK, Springvale, ME, USA) was used as the coating buffer, and second, the samples were transiently acidified and neutralized [47] without first diluting them in DPBS, followed by dilution with Block and Sample 1X Buffer.

### Tyrosine hydroxylase immunohistochemistry

After the dissection described above, the posterior part of the brain was immersed overnight in 4% PFA in PBS at + 4 °C, stored in 20% sucrose in PBS at + 4 °C until freezing in isopentane on dry ice, and stored at − 80 °C. Free-floating coronal sections of 30 µm thickness were cut with a Leica CM3050 cryostat (Leica Biosystems, Wetzlar, Germany) and stored at − 20 °C. TH immunostaining was performed essentially as described by Mijatovic et al. [48], with the exception that biotinylated protein A (prepared using protein A [MP Biomedicals, Santa Ana, CA, USA] and N-hydroxysuccinimido-biotin [Sigma-Aldrich]) was used in place of the secondary antibody to reduce background staining. Optical density across the SNC was measured as described previously [21].

### High-performance liquid chromatography

HPLC was used to determine the striatal tissue concentration of dopamine in separate female C57BL/6J mice. The mice were lesioned with identical 6-OHDA injections into the MFB and subjected to an approximately similar long-term levodopa treatment (12 weeks of daily [Mon–Fri] s.c. injections of 3 mg/kg levodopa and 15 mg/kg benserazide). The collection of samples of dorsal striatum and HPLC were performed as described by Julku et al. [49].

### Data analysis

LID data from the AZD0328 experiment are shown as total dyskinesia scores over time in minutes. Total dyskinesia was calculated as the sum of the three LID subtype scores per time point. For the subtype-specific data see Additional file 1. LID data from the nicotine experiment are shown as weekly severity scores, separately for each subtype, over time in weeks. The weekly dyskinesia score was calculated for each dyskinesia subtype as the integral (area under the curve) of the animal’s time point scores [50], giving the weekly subtype score a theoretical range from 0 to 16. Data points missing due to failed levodopa administration (affecting 2 out of 120 weekly assessments) were interpolated using linear regression on the individual animal’s data. Total weekly dyskinesia scores (Additional file 1) were then calculated as the sum of the weekly subtype scores.

Immunohistochemical data were analyzed with an unpaired Student’s t test. Dyskinesia data were analyzed with two-way repeated measures analyses of variance (ANOVA) [50] with Greenhouse–Geisser correction for violations of the assumption of sphericity followed by Bonferroni’s multiple comparisons tests. BDNF data were expressed as percentage of the mean of the unlesioned hemisphere of the control group and analyzed with two-way ANOVA. Pearson’s correlation coefficients were calculated for the correlation between BDNF levels and weekly dyskinesia scores at the last recording time. All data are represented as either group mean ± SEM or as box plots showing group median, quartiles, range, and distribution. Statistical analyses were performed with IBM SPSS Statistics 24 (IBM, Armonk, NY, USA) with the alpha level set at *P *< 0.05.

## Additional file

**Additional file 1.** All data generated during this study are included within a Microsoft Excel file named Additional file 1.xlsx.
